# Selection and lottery in medical school admissions: who gains and who loses?

**DOI:** 10.15694/mep.2018.0000271.1

**Published:** 2018-12-04

**Authors:** Anouk Wouters, Gerda Croiset, Rashmi A. Kusurkar

**Affiliations:** 1 Amsterdam UMC; 2 Amsterdam UMC

**Keywords:** Admissions, Selection, Lottery, Applicants, Medical Schools, Society, Student Diversity

## Abstract

This article was migrated. The article was marked as recommended.

Concerns related to fairness of medical school admissions through selection have led some scholars to consider selection as an expensive lottery and suggest that lottery may be fairer. This paper considers the issue of selection versus lottery from the perspectives of three groups of stakeholders: 1) applicants, 2) medical schools, and 3) society. This paper contributes to the discussion by addressing advantages and disadvantages of the use of selection and lottery for these stakeholder groups, grounded in the findings from research. Themes that are discussed are reliability and validity issues, perceived influence on selection outcomes and student uptake, effects on student diversity, financial costs, impact on rejected applicants, transparency, and strategic behaviour. For each stakeholder group both lottery and selection yield a combination of advantages and disadvantages, which implies that none of the currently available admissions strategies completely fulfils stakeholders’ needs. Research indicates that selection yields only small gains compared to a lottery procedure, while the student diversity, necessary for serving the increasingly diverse patient population, may be compromised. We argue that society’s needs should drive admissions policies rather than institutional gains, which means that until a selection procedure is developed that does not disadvantage certain types of students, a lottery procedure should be preferred.

## Background

Medical students are admitted to the medical study either through a qualitative selection procedure or a lottery. While in most countries admissions boards have relied on a thorough assessment of applicants’ qualities and developed a variety of selection tools, the Dutch have employed a lottery; a random lottery at first, and a lottery that was weighted for pre-university performance in later years ( Ten Cate, 2007). In this weighted lottery, the chances of students with an outstanding high school grade point average (≥8 out of 10) was 100%, they were therefore granted direct access to the medical study. Finally, the need for control and a perceived lack of fairness instigated a gradual change from lottery-based to selection-based admissions. Globally, however, concerns related to fairness and equity have led some scholars to consider selection as an ‘expensive lottery’ ( Norman, 2004; Groves, Gordon and Ryan, 2007) and inefficient in selecting the diverse future workforce necessary for meeting the needs of a changing society ( General Medical Council, 2009). They suggest that a real lottery might be preferable after all ( Benbassat and Baumal, 2007; Brown and Lilford, 2008). This paper contributes to the discussion about the controversial topic of medical school admissions by reflecting on the advantages and disadvantages of using lottery or selection procedures for different stakeholders (i.e. applicants, medical schools, and society), so that policy makers can make an informed choice for their own situation. We, in no way, claim to be exhaustive with this article and encourage admissions boards and scholars to be sensitive to any possible other aspects, especially relevant to their own context.

## Applicants

The interests of applicants in medical school admissions pertain to perceived fairness and transparency, perceived influence on admissions outcomes and perceived chances of success, impact of rejections, and strategic behaviour.

It is widely acknowledged that selection should be fair and transparent ( Benbassat and Baumal, 2007; Patterson *et al.*, 2018). From the applicants’ perspectives fairness and transparency means that they can understand what is needed to be successful in selection and that they do not feel hindered in achieving this. This way, applicants can feel in control of their own fate ( Wouters *et al.*, 2016). In practice, however, designing a selection procedure that is completely transparent and fair to all applicants proves to be a challenge. How applicants perceive their influence on admissions outcomes can differ depending on their socio-economic background and their access to preparatory activities ( Greenhalgh, Seyan and Boynton, 2004; Rao and Flores, 2007; Freeman *et al.*, 2015; Southgate, Kelly and Symonds, 2015). Applicants from ‘non-traditional’ backgrounds more often underestimate their chances in selection, which can cause them to be wrongfully discouraged from applying ( Wouters *et al.*, 2017a). In addition, research has shown that students from underrepresented sociodemographic groups *do* have smaller chances to be offered a place in medical school due to biased selection procedures ( Mcmanus *et al.*, 1995; McManus, Aneez and Marie, 1998; Laurence *et al.*, 2010; Puddey *et al.*, 2011; Tiffin, Dowell and McLachlan, 2012; O’Neill *et al.*, 2013; Griffin and Hu, 2015; Stegers-Jager *et al.*, 2015; Mathers, Sitch and Parry, 2016; Steven *et al.*, 2016). The consequences of these mechanisms extend beyond the stakeholder group of applicants, and are further discussed below. Being transparent about the selection criteria could be an important tool in counteracting this mechanism. However, if medical schools reveal their exact scoring method, this may interfere with the measurement of the instrument and incite ‘faking good’ behaviour among applicants ( Griffin and Wilson, 2012). Applicants may game the system by behaving according to what they understand is expected of them instead of showing their true nature, undermining the purpose of the assessment tool. Such strategic behaviour also reflected in applicants’ medical school choices (which can be only one per year in the Dutch setting), which is dominated by the type of selection procedure employed ( Wouters *et al.*, 2017c). A lottery procedure, either weighted for previous academic achievement or not, is very transparent. Especially in a completely random lottery, all applicants meeting the minimum entrance requirements know they have equal opportunities. However, applicants have no opportunity to exert influence on their admissions chances in a lottery.

Another aspect of relevance to applicants is the impact of being rejected. This area is currently understudied. A rejection based on impersonal grounds (in a lottery) can be expected to have less impact on applicants’ self-esteem than a rejection based on personal grounds (in a selection procedure, especially when personal attributes are assessed) ( Benbassat and Baumal, 2007). Experiencing rejection as personal failure can elicit feelings of shame among applicants, which is associated with decreased well-being ( Bynum and Artino, 2018). Success in selection, on the other hand, is experienced as a confirmation of students’ suitability for medical study and can stimulate feelings of pride, and temporarily boost students’ motivation ( Wouters *et al.*, 2016).

In conclusion, advantages of selection for applicants include the perceived feeling of having influence on one’s admission, positive self-esteem on being selected, while disadvantages include negative effects of a personal rejection, the incitement of strategic behaviour, perceived barriers, and the lack of transparency. Advantages of lottery include transparency, equal opportunities and no personal rejection (just bad luck), while disadvantages include no influence on admissions outcomes.

## Medical schools

The interests of medical schools in medical school admissions pertain to influence on student uptake, the design of effective admissions procedures, and financial costs.

Qualitative selection procedures provide medical schools with the opportunity to exert influence on which students get enrolled, while a lottery procedure does not. Medical school admissions boards strive to select the most suitable students from the applicant pool. Ideally, a thorough assessment of applicants results in the selection of a group of highly motivated students who will perform their best in medical study and practice. Generally, however, identifying clear outcome measures has proven difficult, and evidence for the predictive validity of different selection tools is weak ( Benbassat and Baumal, 2007; Cleland *et al.*, 2012; Patterson *et al.*, 2016).

A great variety of performance outcomes is considered in research investigating the differences between selected and lottery-admitted students and the evidence is inconclusive. While in some studies better academic performance, such as higher grades, lower dropout and better professional behaviour among selected students, is reported, in many studies, differences are often not found or are not significant. Moreover, differences between the admission groups seem to decline throughout the years. In some studies, better outcomes were found among selection participants in comparison with students who had only participated in the lottery procedure ( Stegers-Jager, 2018). The one consistent finding is that students from the 100%-chance lottery category (highest pu-GPA) perform best in both pre-clinical and clinical medical education. See table 1 for an overview of studies comparing lottery and selection. Comparable findings between selected and rejected (but admitted to another medical school) students have also been reported in Canada ( Kulatunga-Moruzi and Norman, 2002).

For interpretive reasons it is important to understand the context of Dutch admissions. Dutch secondary education is divided into three main streams: one stream to prepare students for vocational training, one to prepare them for universities of applied sciences and one stream to prepare students for university (pre-university secondary education). Every student that completes the six-year pre-university education is eligible for enrolling in a university study and can therefore be expected to be able to successfully complete their studies. For the medical study, successful completion of four science subjects (chemistry, biology, physics and math) at pre-university level is required. All students with other educational backgrounds have to show proof that they meet similar educational levels. Students that meet these prerequisites can apply for the medical study. It may therefore not be surprising that differences between the two admissions groups are small and do not always reach significance.

Student motivation and engagement have also been compared between selected and lottery-admitted students. Differences between the groups mainly pertained to strength of motivation, in which selected students had the highest scores ( Hulsman *et al.*, 2007; Kusurkar *et al.*, 2010; Wouters *et al.*, 2016; Wouters *et al.*, 2017b) except in one study, where differences did not reach significance ( Nieuwhof *et al.*, 2004). More importantly, the quality of motivation and student engagement did not seem to differ between selected and lottery-admitted students ( Wouters *et al.*, 2016; Wouters *et al.*, 2017b). Only one study found better quality of motivation among selected students in comparison with lottery-admitted students ( Kusurkar *et al.*, 2013b) ( Table 2).

Researchers have applied the Taylor-Russell model to evaluate the practical usefulness of selection and have drawn similar conclusions ( Niessen and Meijer, 2016). The Taylor-Russell model can be used to determine the success ratio of selection. The success ratio is calculated based on the base rate, selection ratio and the predictive validity of the selection procedure. Here, base rate means the expected success rate without applying selection, and selection ratio means the percentage of students that will be selected. A small effect of selection can be expected when either the base rate or the selection ratio is high. For the Dutch context, calculations showed that with a base rate of around 0.80 and a selection ratio of around 0.60, the success rate of increases with 1.8% when selection is applied. This increase from 81.3% to 83.1% corresponds with a gain of around 6 successful students at each medical school (with a total number of 2785 places in eight different medical schools). In sum, the gains in selection seem to be small compared to lottery, and some researchers have advocated the use of a lottery system after certain academic standards have been met ( Benbassat and Baumal, 2007; Hubbeling, 2017).

**Table 1.** Overview of findings regarding selection and lottery in pre-clinical and clinical years of medical education

Outcome measure	Findings
Year-1 performance / course credits	GPA: No differences ( Hulsman *et al.*, 2007; Wouters *et al.*, 2017b) for 3 out of 4 cohorts ( Urlings-Strop *et al.*, 2009; Lucieer *et al.*, 2015) Selected > lottery-admitted ( Schripsema *et al.*, 2014; Schripsema *et al.*, 2017; Schreurs *et al.*, 2018b), including rejected lottery-admitted ( de Visser *et al.*, 2017); for 1 out of 4 cohorts in 1 out of 4 study years ( Urlings-Strop *et al.*, 2009) Progress test: Selected > rejected lottery-admitted ( Schreurs *et al.*, 2018b) OSCE: percentage fail (/non-fail): no differences ( Schreurs *et al.*, 2018b) percentage good (/non-good): selected > rejected lottery-admitted ( Schreurs *et al.*, 2018b) Course credits: No differences ( Hulsman *et al.*, 2007; Urlings-Strop *et al.*, 2009; Wouters *et al.*, 2017b); no differences between selected and rejected lottery-admitted ( de Visser *et al.*, 2017; Schripsema *et al.*, 2017) Selected and rejected lottery-admitted > lottery-admitted ( Schripsema *et al.,* (2014)( de Visser *et al.*, 2017) Selected > rejected lottery-admitted ( Schripsema *et al.*, 2017) Selected > lottery (after including early medical school performance this did not remain) ( Stegers-Jager *et al.*, 2015)
Year-2 performance / course credits	GPA: No differences between selected and rejected lottery-admitted ( Schreurs *et al.*, 2018b) Progress test: Selected > rejected lottery-admitted ( Schreurs *et al.*, 2018b) OSCE: percentage fail (/non-fail): no differences ( Schreurs *et al.*, 2018b) percentage good (/non-good): selected > rejected lottery-admitted ( Schreurs *et al.*, 2018b) Course credits: No differences between selected and lottery-admitted ( de Visser *et al.*, 2017) Selected and rejected lottery-admitted > lottery-admitted ( Schripsema *et al.*, 2014) Selected > rejected lottery-admitted ( de Visser *et al.*, 2017)
Year-3 performance / course credits	Cognitive tests: Critical Appraisal of a Topic test: no differences for ( Schreurs *et al.*, 2018b) Percentage fail (/non-fail): selected < rejected lottery-admitted ( Schreurs *et al.*, 2018b) Percentage excellent (/non-excellent): no differences ( Schreurs *et al.*, 2018b) Progress test: No differences ( Schreurs *et al.*, 2018b) OSCE: No differences ( Lucieer *et al.*, 2015) Selected > rejected lottery-admitted ( Schreurs *et al.*, 2018b) Course credits: No differences ( Schripsema *et al.*, 2014) between selected and lottery-admitted ( de Visser *et al.*, 2017) Selected > rejected lottery-admitted ( de Visser *et al.*, 2017)
Year-4 clerkships	No differences between selected and lottery-admitted ( Wouters *et al.*, 2017b)Selection participants > non-participants ( Wouters *et al.*, 2017b)
Interpersonal outcomes	Professionalism : No differences ( Schripsema *et al.*, 2017; Schreurs *et al.*, 2018b)Selected > lottery-admitted, including rejected lottery-admitted ( Schripsema *et al.*, 2014) Consulting and reflecting skills: Percentage fail (/non-fail): No differences in Y1 and Y2 ( Schreurs *et al.*, 2018b) Percentage good (/non-good): selected > rejected lottery-admitted in Y1 and Y3, no differences in Y2 ( Schreurs *et al.*, 2018b)
Bachelor completion in 3 years	No differences ( Lucieer *et al.*, 2015; de Visser *et al.*, 2017; Schreurs *et al.*, 2018b)
Bachelor completion in 4 years	No differences ( Lucieer *et al.*, 2015)
Dropout	No differences between selected and rejected lottery-admitted students ( de Visser *et al.*, 2017; Schreurs *et al.*, 2018b) Selected < lottery-admitted ( Urlings-Strop *et al.*, 2009; Urlings-Strop *et al.*, 2011; de Visser *et al.*, 2017)

**Table 2.** Overview of research on admissions groups and motivation and engagement

Authors	Findings
Nieuwhof *et al.*, 2004	No differences with regards to the strength of motivation of selected and lottery-admitted students
Hulsman *et al.*, 2007	Higher strength of motivation among selected students in comparison to lottery-admitted students
Kusurkar *et al.*, 2010	Higher strength of motivation among selected students in comparison to lottery-admitted students
Kusurkar *et al.*, 2013a	Higher autonomous motivation among selected students in comparison to lottery-admitted students
Wouters *et al.*, 2016	Higher strength of motivation among selected students in comparison to lottery-admitted and top pu-GPA studentsNo differences with regard to the quality of motivationHigher strength of motivation, autonomous motivation and controlled motivation among recently selected students in comparison with non-selected students and students who were selected longer ago
Wouters *et al.*, 2017b	Higher strength of motivation among selected students in comparison with top pu-GPA students.No differences with regards to quality of motivationNo differences with regards to engagement

Although the usage of evidence-based selection methods (e.g. MMIs and SJTs) has increased over the past years, methods that are not supported by research evidence (e.g., reference letters and motivation assessments) are still widely used ( Patterson *et al.*, 2016). Support for the reliability and validity of selection tools currently in use is mixed and appears to be strongly influenced by the context in which the tools are used ( Cleland *et al.*, 2012; Patterson *et al.*, 2016; Patterson *et al.*, 2018). A phenomenon of danger to the validity and reliability of selection tools assessing personal qualities is the previously described ‘faking good’ behaviour ( Mueller-Hanson, Heggestad and Thornton, 2003; Griffin and Wilson, 2012).

There is no consensus on what should be achieved with selection and a lack of clear outcome measures to assess to what extent goals are achieved. Do we wish to select students who will perform well in the pre-clinical phase of medical education, in the clinical phase, or even in practice ( Stegers-Jager, 2018)? Increasingly incorporating assessment of attributes in selection that are considered relevant for identifying ‘the good doctor’ suggests that selection has long-term effects, whereas the role of the subsequent educational programme is to train all students for becoming ‘good doctors’. In addition, students’ professional development continues throughout their professional career. With that, it is recognized that there is no such thing as one type of ‘good doctor’ ( Hurwitz and Vass, 2002; Hubbeling, 2018). Different medical professions call for different qualities. With a lottery procedure no long-term effects with regards to the quality of the student population are implied and it allows for students with different qualities to become trained in the medical programme.

The use of a (random) lottery is inherently cheaper than a qualitative, usually multi-method, selection procedure. However, these strategies may yield different financial benefits depending on how well a procedure is able to yield a student population that accounts for financial returns. Schreurs et al. were the first to make a cost-benefit comparison of selection and lottery ( Schreurs *et al.*, 2018a). They found that, although implementing a selection procedure is costly, its use can turn out beneficial in terms of reduction of costs associated with dropout and repetition of blocks and OSCEs in the Bachelor phase of the medical study. A decrease in dropout due to a qualitative selection procedure turned out to yield the biggest gains. Further research is needed to build evidence for the cost-effectiveness of selection over a lottery procedure.

In conclusion, gains from selection for medical schools include (possible financial returns due to) better achieving students, and self-selection of less suitable applicants, while losses pertain to high costs involved in developing and performing selection procedures, the difficulty of designing reliable and valid procedures, and undermining of the educational programme (pretends to have long-term effects). Advantages of a lottery include low costs, it respects the role of the educational programme, and when certain educational standards are met students are generally able to perform well in their studies ( Hubbeling, 2018). A disadvantage is that medical schools have no influence on which students are admitted.

## Society

The interests of society concern creating a student and professional workforce that meets the needs of the society they will serve ( General Medical Council, 2009). This includes admitting a diverse group of students, and admitting a group of students aspiring jobs in understaffed professional domains and geographical areas. Furthermore social accountability can include stimulating an informed choice among future students.

Society is becoming increasingly diverse and older and a shift in workforce planning implies that more health care professionals are needed in primary care, and less in tertiary care ( McPake, Auraújo Correia and Gillian, 2017). In reality, however, the majority of medical students aspire for a career in specialty care ( Compton *et al.*, 2008). Whereas with a completely random lottery admissions boards can exert no influence on who gets admitted, the amount of influence increases with a weighted lottery and is maximized with a qualitative selection procedure. Admissions boards can use their influence to meet their respective goals by both showcasing what types of students they are looking for and emphasizing what is valued, and by attempting to identify those students in selection. Currently, all applicants to a particular medical school are generally assessed based on the same criteria. This provides no room for differentiating according to the healthcare needs.

What admissions boards are looking for and strive for can predominantly be categorized as excellence and top performance. However, the value of diversity in medical education, or attracting a diverse group of applicants, for meeting the needs of the diverse society, is usually not put forth as part of the aims of selection ( Alexander *et al.*, 2017). If the medical workforce shows a good representation of the diversity in the patient population, every individual can be provided the best possible healthcare for ( Morgan *et al.*, 2016). Therefore, medical schools have the responsibility to generate a student population that is a reflection of the society it will serve in the future ( General Medical Council, 2009; Frenk *et al.*, 2010). There are concerns, however, that student diversity may be compromised due to selection ( Grafton-Clarke, 2016; Wouters, 2017a; A. Wouters *et al.*, 2017a). In particular, students from ethnic minority backgrounds ( Rao and Flores, 2007; Young *et al.*, 2012), students without a medical family background ( McManus and Richards, 1984; Heath, Stoddart and Green, 2002; Simmenroth-Nayda and Gorlich, 2015; Wouters *et al.*, 2017a), lower socioeconomic status students ( Heath, Stoddart and Green, 2002; Ferguson *et al.*, 2012; Young *et al.*, 2012), and students who are first in family to go to university ( Vaglum, Wiers-Jenssen and Ekeberg, 1999; Heath, Stoddart and Green, 2002; Gasiorowski, Rudowicz and Safranow, 2015) are underrepresented in medical education.

A first explanation pertains to biased selection procedures ( Mcmanus *et al.*, 1995; Puddey *et al.*, 2011; Cleland *et al.*, 2012). Furthermore, self-selection among those students has been suggested as a possible cause for their underrepresentation ( Stegers-Jager *et al.*, 2015). Self-selection occurs when students decide to refrain from applying to medical study. For example, research has indicated that having a medical doctor as a parent makes it easier to gain information about the medical profession, as well as acquiring internships in healthcare, which is often part of the selection criteria ( Wouters *et al.*, 2017a). Also, commercial training agencies respond to applicants’ fears of not being able to fulfil their lifelong desire of studying medicine by offering expensive preparatory trainings, which are not accessible to all applicants. Although evidence for the effectiveness of such trainings is inconclusive ( Griffin *et al.*, 2008; Laurence *et al.*, 2013), applicants grab every opportunity to enhance their chances of success in selection.

These mechanisms imply that the currently used criteria contribute towards the inequality in access to medical education. Widening participation/access efforts are enacted to remove barriers, compensate for disadvantages and encourage underrepresented students into higher education. Such efforts, however, vary in the extent to which they are successful ( Cleland *et al.*, 2012; Grafton-Clarke, 2016). An unweighted lottery procedure is free of such socio-cultural barriers, which takes away the need for widening access initiatives.

Selection could be useful for encouraging applicants to gather information about the medical study and reflect on their study choice which can stimulate a well-informed decision on applying ( Wouters *et al.*, 2014; Niessen and Meijer, 2016). When students have to reflect on why they apply for (a certain) medical school, they are stimulated to study the information provided by medical schools to substantiate their statements ( Wouters *et al.*, 2014; de Visser *et al.*, 2018). However, students with highly educated (medical) parents, have been found to be able to receive more practical help in making a study choice ( Wouters *et al.*, 2017a). For participation in a lottery procedure, no substantive preparations are required.

In conclusion, from the societal perspective, advantages of selection include the opportunity to select a diverse future workforce according to society’s needs (which is currently not used optimally), while disadvantages include a decrease in diversity, and self-selection of suitable applicants. An advantage of lottery pertains to better diversity, while it is not possible to actively influence the student uptake according to society’s needs.

## Conclusions

The choice for an admissions strategy should be a parsimonious one based on a proper weighing of the consequences. For each group of stakeholders both lottery and selection procedures yield advantages and disadvantages. This implies that it is difficult to completely satisfy either one of the stakeholder groups with the currently available admissions strategies. It also suggests that it is impossible to satisfy all stakeholder groups simultaneously. Perhaps the solution can be found in the previously employed combination of lottery and selection instead of choosing one over the other. This way selection accounts for perceived face validity and fairness, while a lottery serves to reduce the costs and the damage that selection may bring to diversity. The primary author, AW, has been rejected in the lottery procedure thrice. But after completing a PhD on this topic, which made it clear that selection has only a few advantages over lottery and has a negative influence on applicant diversity, she pleads for the reintroduction of an unweighted lottery in the Netherlands ( Wouters, 2017a; Wouters, 2017b). We argue that society’s needs should drive admissions policies rather than institutional gains. Unless there is evidence that a particular selection procedure in a specific context is not biased based on improper grounds, a lottery seems preferable over selection procedures ( Hubbeling, 2018). It appears that in the Dutch context a relatively inexpensive and fair (in terms of equity) procedure that yielded a well-performing, motivated student population was exchanged for a more expensive procedure which seems to disadvantage students that are underrepresented in medical education.

## Take Home Messages

• We considered the interests of three stakeholder groups in lottery and selection in medical school admissions , i.e. applicants, medical schools and society.
• For each stakeholder group both lottery and selection yield a combination of advantages and disadvantages, which implies that none of the currently available admissions strategies completely fulfils stakeholders’ needs.
• Compared to a lottery procedure, selection procedures seem to yield small gains, while student diversity, necessary for serving the increasingly diverse patient population, may be hampered.
• We argue that society’s needs should drive admissions policies rather than institutional gains.

## Notes On Contributors

AW is a psychologist by training and completed a PhD in medical education, focusing on selection and motivation for the medical study. ORCID ID: https://orcid.org/0000-0002-6806-7573

GC is a professor in medical education at VUmc School of Medical Sciences, and Dean of Education and Training at University Medical Center Groningen.

RAK is a medical doctor by training, an assistant professor and the head of the department of Research in Education at VUmc School of Medical Sciences. ORCID ID: https://orcid.org/0000-0002-9382-0379
