# Supplementary material for: Selection and lottery in medical school admissions: who gains and who loses?
Source: MedEdPublish (2016). 2018 Dec 4;7:271. [Version 1] doi: 10.15694/mep.2018.0000271.1 (PMC10711990; doi:10.15694/mep.2018.0000271.1)
Supplement: Peer review history

# Reviewer report: Reviewer response for version 1

**DOI:** 10.21956/mep.19730.r29555

**Published:** 2018-12-11

**Authors:** Joy Cox

**Affiliations:** 1 American University of Antigua

This review has been migrated. The reviewer awarded 4 stars out of 5 Thank you for sharing this informative and significant opinion piece debating the differences between selection and lottery-admitted medical students. The arguments are outlining the advantages and disadvantages of both approaches from the perspective of the applicant, medical schools and society are valid. Although no conclusive decision was conveyed about which approach is better, the paper opens the door to a topic which needs to be discussed further and it provides readers, particularly stakeholders in the admissions process, with ‘food for thought’. Are medical schools using a criteria-based selection process compromising student diversity required to meet society’s health care needs? Will the lottery-admitted medical students be less motivated and engaged that students who were selected? I would recommend this paper to all medical educators and health care professionals working in student admissions.

Reviewer Expertise:

NA

No decision status is available

# Reviewer report: Reviewer response for version 1

**DOI:** 10.21956/mep.19730.r29556

**Published:** 2018-12-06

**Authors:** Sateesh Babu Arja

**Affiliations:** 1 Avalon University School of Medicine

This review has been migrated. The reviewer awarded 4 stars out of 5 I read this paper with great interest. It is very well written and authors have taken no side towards either selection or lottery process. Even though this paper is inconclusive, authors have clearly explained the advantages and disadvantages associated with both the selection process and lottery. The only concern with the lottery process that I have is how can we assess the personal characteristics or traits of the candidate? However, these attributes are unstable and medical education is intended to teach these attributes. Authors quoted "Advantages of lottery include transparency, equal opportunities and no personal rejection (just bad luck)". It is not just bad luck. It will have a huge impact on the applicant. But I would like to commend the authors for presenting such an important and controversial topic in the admissions process of medical schools. The literature review and references are very relevant and updated. This paper is useful for the faculty members involved in the admission process.

Reviewer Expertise:

NA

No decision status is available

# Reviewer report: Reviewer response for version 1

**DOI:** 10.21956/mep.19730.r29554

**Published:** 2018-12-04

**Authors:** Trevor Gibbs

**Affiliations:** 1 AMEE

This review has been migrated. The reviewer awarded 4 stars out of 5 Thank you for asking me to review this paper, which covers an important yet unresolved question in medical and healthcare education- how to select our students who will become fit-for-purpose professionals. The authors chose an interesting approach, and -, although there were a few areas that were difficult to understand, I think presented their case clearly. At the end of the day, I don't feel that there was any clear winner between the lottery approach and the criteria-based selection approach, which probably reflects why after all the previous research, we are still writing papers in this area. Perhaps the paper also shows that we are very dependent on what Schools / Institutions believe rather than what society wants.Although somewhat inconclusive I do think that the paper should be read by those involved in the process of student selection- the references mentioned are particularly relevant and cover many years of study

Reviewer Expertise:

NA

No decision status is available
